# Epitaxy of (11–22) AlN Films on a Sputtered Buffer Layer with Different Annealing Temperatures via Hydride Vapour Phase Epitaxy

**DOI:** 10.3390/ma17020327

**Published:** 2024-01-09

**Authors:** Xuejun Yan, Maosong Sun, Jianli Ji, Zhuokun He, Jicai Zhang, Wenhong Sun

**Affiliations:** 1 Research Center for Optoelectronics Materials and Devices, School of Physical Science and Technology, Guangxi University, Nanning 530004, China; xjuny100@163.com (X.Y.); 2023700080@buct.edu.cn (M.S.); jijianli2021@163.com (J.J.); hzkgmy@hotmail.com (Z.H.); 2 College of Mathematics and Physics, Beijing University of Chemical Technology, Beijing 100029, China; 3 State Key Laboratory of Chemical Resource Engineering, Beijing University of Chemical Technology, Beijing 100029, China; 4 State Key Laboratory of Featured Metal Materials and Life-Cycle Safety for Composite Structures, Nanning 530004, China; 5 Guangxi Key Laboratory of Processing for Nonferrous Metals and Featured Materials, Guangxi University, Nanning 530004, China

**Keywords:** semipolar AlN, hydride vapour phase epitaxy, HTA, magnetron sputtering, regrowth

## Abstract

AlN epilayers were grown on magnetron-sputtered (MS) (11–22) AlN buffers on *m*-plane sapphire substrates at 1450 °C via hydride vapour phase epitaxy (HVPE). The MS buffers were annealed at high temperatures of 1400–1600 °C. All the samples were characterised using X-ray diffraction, atomic force microscopy, scanning electron microscope and Raman spectrometry. The crystal quality of epilayers regrown by HVPE was improved significantly compared to that of the MS counterpart. With an increasing annealing temperature, the crystal quality of both MS buffers and AlN epilayers measured along [11–23] and [1–100] improved first and then decreased, maybe due to the decomposition of MS buffers, while the corresponding anisotropy along the two directions decreased first and then increased. The optimum quality of the AlN epilayer was obtained at the annealing temperature of around 1500 °C. In addition, it was found that the anisotropy for the epilayers decreased significantly compared to that of annealed MS buffers when the annealing temperature was below 1500 °C.

## 1. Introduction

Aluminium nitride (AlN) has a great potential application in the field of deep ultraviolet (UV) optoelectronic devices and high-power, high-frequency electronic devices due to its wide band gap and outstanding material properties [1,2]. However, there is strong spontaneous and piezoelectric polarisation along the AlN [0001] direction. The optoelectronic devices grown along this direction suffer from polarised electric fields, which reduce the overlap of wave functions of the electron and hole and decrease the internal quantum luminescence efficiency. Nonpolar AlN (such as (11–20) and (10–10)) is used as a substrate or template for optoelectronic devices [3,4]. Due to the strong anisotropy of nonpolar AlN, it is difficult to grow high-quality nonpolar AlN with a smooth surface [5]. Therefore, semi-polar AlN, such as (10–11), (10–13) and (11–22) planes, is a good choice, which could significantly suppress the effect of the polarised electric fields [6]. Over the last decade, controlling the phase structure and improving the crystal quality of semipolar AlN on sapphire has been studied intensively; Mogilatenko et al. analysed the formation of semipolar AlN planes with different orientations on interstitial sapphire [7]. Despite the advances achieved, the presence of dislocations and layer defects remains a major challenge in the development of semipolar AlN films.

Due to the lack of large-area and low-cost native substrates, AlN-based UV devices have generally been grown on foreign substrates such as sapphire and SiC [8,9,10,11,12,13,14]. However, the growth of high-quality AlN is extremely difficult because of the low migration of Al adatoms on the surface, which always leads to the columnar growth mode and results in a rough surface. To improve the crystal quality of the AlN epilayer, many methods have been developed, such as high-temperature growth [15,16,17,18], nano-patterned sapphire [19,20], sapphire nitridation pretreatment [21] and so on. Recently, high-quality AlN templates grown by metal-organic chemical vapour deposition (MOCVD) and hydride vapour phase epitaxy (HVPE) using magnetron-sputtered (MS) AlN on sapphire as a buffer layer have been reported [22,23,24,25,26,27,28,29]. Relevant studies on the growth of high-quality AlN thin films in the combination of HVPE + MS are shown in Table 1 below. It can be seen that the studies related to this method of epitaxial AlN films are all AlN films on the polar (0002) side, and there are fewer studies related to semipolar AlN films.

Magnetron sputtering is an important low-cost technique for depositing AlN-thin films at a low temperature. However, the sputtered AlN thin films often contains high defect densities. The crystal quality of such films could be improved by a high-temperature annealing process [27,35,36,37,38,39,40]. Even though the quality was improved, the thickness of the AlN film was too thin to be used directly for device epitaxy. Generally, a thick AlN film needed to be grown on such a sputtered thin film before the device epitaxy. In this work, a thickness of 200 nm AlN was sputtered on m-sapphire at 400 °C and annealed at a high temperature (HTA), which is highly stable and reproducible. Then, HVPE was used to grow (11–22) the AlN epilayer on such MS AlN buffers. The influence of the annealing temperature of MS AlN buffers on the AlN epilayer was investigated. It was found that the increase in annealing temperature favoured the improvement of the crystal quality and the anisotropy. However, a too-high annealing temperature damaged the AlN epilayer due to the decomposition of the MS AlN buffer.

## 2. Materials and Methods

A 200 nm thick magnetron-sputtered (MS) AlN buffer layer was deposited on an m-sapphire substrate with a sputtering temperature of 400 °C. The MS AlN films were annealed in a high-temperature tubular annealing furnace (1750x–) under a 150 Torr nitrogen atmosphere at 1400, 1500, 1550 and 1600 °C for 30 min, which were named B1, C1, D1 and E1, respectively. The AlN epilayers were grown using a hydride vapour phase epitaxy (HVPE) at a temperature of 1450 °C on the above annealed MS AlN films. The thickness of the AlN epilayer was around 2.4 µm. For reference, the AlN epilayer on sputtered AlN thin films without an annealing process (named A1) was also grown using HVPE, marked as A. Table 2 lists the names and characteristics of the samples used in this work.

The surface morphology of the samples was studied using an atomic force microscope (AFM) on Bruker FASTSCOULD (Bruker, Beijing, China) and scanning electron microscopy (SEM) on MAIA3 Tescan (ZEISS, Beijing, China), and the quality of the AlN film was characterised using X-ray diffraction (XRD) on PANalytical X’Pert3MRD (MalvernPanalytical, Beijing, China). Raman spectra were measured using a Labram HR Evolution (Horiba, London, England) with a laser wavelength of 532 nm.

## 3. Results and Discussion

**Figure 1:**
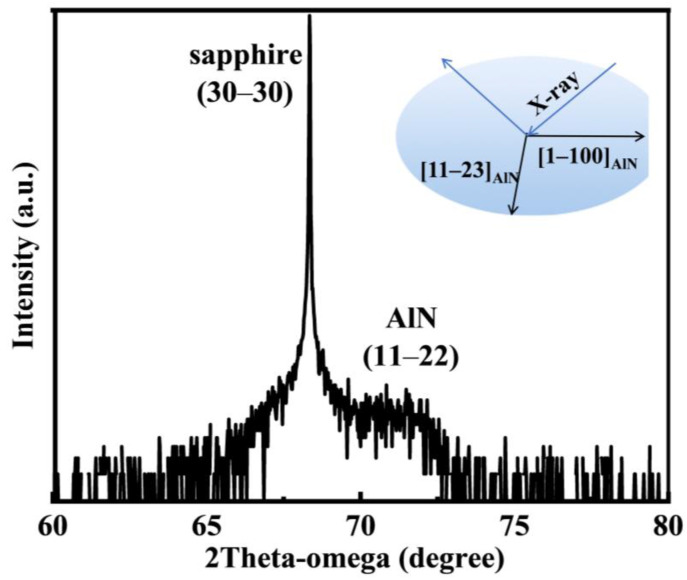
The 2theta-omega scanning curves of semipolar AlN films (the inset shows the 2theta-omega setup).

The overall crystal quality of the AlN epitaxial layer was investigated using XRD. The FWHMs of the XRC of the AlN (11–22) facets were obtained at two azimuthal angles, revealing the anisotropic characteristics of the semipolar AlN epitaxial layer. Figure 1 shows the 2theta–omega scanning curves of the sputtered semipolar (11–22) AlN films (where the inset was an inset plot showing the 2theta-omega setting). As shown in Figure 1, the AlN epitaxial layer exhibits the in-plane orientation relationship of (11–22) AlN//(10–10) sapphire, and it can be noticed that the sputtered semipolar AlN film has a single orientation and the only presence of the (11–22) plane.

**Figure 2:**
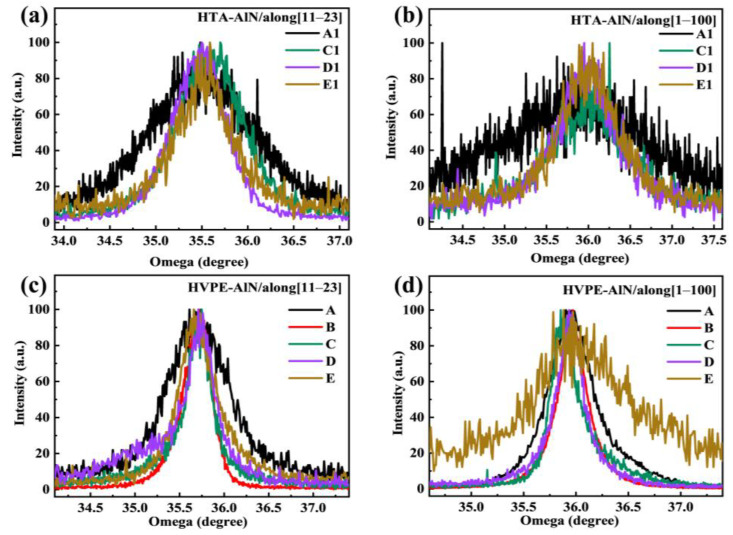
The (11–22) XRD rocking curves along [11–23] and [1–100] for (**a**) and (**b**) HTA-AlN, (**c**) and (**d**) HVPE-AlN of the epilayers.

**Figure 3:**
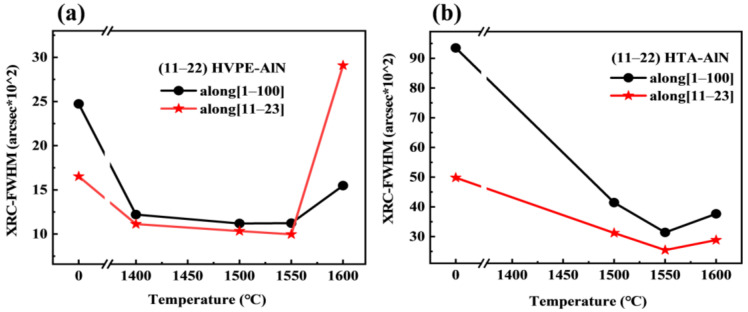
The dependence of FWHM on the annealing temperature for (**a**) epilayers and (**b**) MS buffers.

Figure 2 shows the (11–22) XRD rocking curves along [11–23] and [1–100] for all the epilayers, respectively. Figure 2a,b show the wobble curves in both directions for the MS buffer layer. It was found that the line width of the wobble curves decreased with increasing annealing temperature, while it increased at 1600 °C, where the AlN buffer layer deteriorated the film quality after annealing at 1400 °C, making it impossible to test the rocking curve. Figure 2c,d show the wobble curves in both directions for HVPE-AlN films, and it was found that the line width of the wobble curves decreased with increasing annealing temperature and increased at 1600 °C, which is in accordance with the change in the corresponding buffer layer, indicating that the quality of the re-grown AlN films is affected by the buffer layer. The dependence of the full width at half maximum (FWHM) on the annealing temperature of the MS AlN buffers is shown in Figure 3a. The quality of epilayers grown on the annealed MS buffers at a high temperature below 1550 °C could be improved compared with that on the as-grown buffer. However, the quality of epilayers began to decrease when the annealed temperature was 1600 °C. The corresponding FWHM of the MS buffer under annealing temperatures between 1500 °C and 1600 °C is shown in Figure 3b. The annealing process could increase the crystal quality of MS buffers significantly. At a high temperature of 1600 °C, the quality began to worsen compared to those of other annealed samples, which might be due to the decomposition of MS buffers annealed at such a high temperature. Figure 3a,b showed that the half-peaks of the HVPE-grown AlN films are smaller than those of the corresponding MS buffer layers, indicating that the quality of the regrown HVPE-AlN films is further improved, with a lower density of defective dislocations in the films and improved crystal quality.

**Figure 4:**
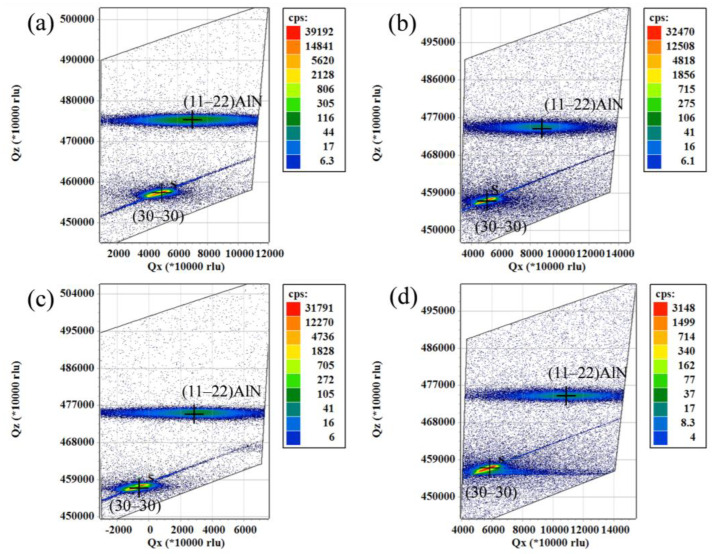
(**a**) Sample B, (**b**) sample C, (**c**) sample D and (**d**) and sample E were the inverse easy space maps of re-grown AlN films treated using high temperature annealing. (Where + is the strongest point in the centre of the sapphire/AlN.).

The reciprocal space maps (RSMs) were measured along the [11–23] direction to study the strain relaxation of the (11–22) AlN layer with respect to the m-plane sapphire, as shown in Figure 4. It was found that the angle between the elongation direction of the AlN (11–22) lattice point in the (11–22) AlN film XRD mapping map and the elongation direction of the (30–30) lattice point of the sapphire substrate was exactly 58 degrees, and the shift in this direction was completely influenced by the layer error within the (11–22) plane, which lay in the direction of the c-plane, resulting in an inverse spatial mapping shift in the stretching direction. Along the c direction, there was the widening of the (11–22) lattice point due to the internal defect stress. The misalignment of the AlN reflection centre along [11–23] with that of the substrate was found in Figure 4, which suggests that this layer is misaligned around the [1–100] axis, i.e., there was a certain tilt angle between the [11–23] AlN and the [0001] sapphire, and the tilt angles of the samples were 1.9°, 3.3°, 2.9° and 4.5°, respectively, and this epitaxial tilt was attributed to (11–22) strain relaxation and lattice tilting in nitrides [41,42].

**Figure 5:**
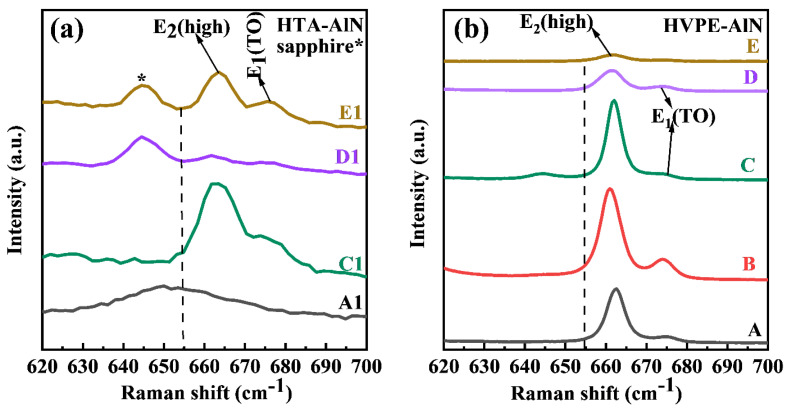
The Raman spectra of all the epilayers. (**a**) HTA–AlN, (**b**) HVPE–AlN.

Figure 5 shows the Raman spectra of all the epilayers. The strain-free position for AlN is marked by the dotted line. For AlN films, the frequency shift of the E_2_(high) peak in Raman spectra is usually used to reflect the in-plane stresses in AlN films, and since the E_2_(high) peak position is highly sensitive to biaxial strains in the c-plane, the difference between the E_2_(high) peak position of the samples and that of the unstressed AlN samples (657.4 cm^−1^) indicates the amount of residual stress. This is a surface feature of in-plane compressive stress if the peak position shows a redshift in the Raman shift, and conversely, if the peak position is blue-shifted. The peak position of E_2_(high) of the sputtered AlN film, located at 651.41 cm^−1^, indicates that the initial stress of the sputtered AlN film is in the state of strong tensile stress. After the high-temperature annealing of the AlN films, the peak position of E_2_(high) for AlN films shows a blue-shift phenomenon, as shown in Figure 5a. It can be seen that the peak positions of phonon peaks of AlN films after high-temperature annealing are all larger than 657.4 cm^−1^, indicating that the stresses of the semipolar films are changed from the strong tensile stress to strong compressive stress via high-temperature annealing treatment, which prevents the subsequent growth of the films from cracking. It inhibits the possibility of cracking in the subsequent growth of the films. The E_2_(high) peak of the HVPE-AlN film is shown in Figure 5b at around 661 cm^−1^, which indicates that all epitaxial layers are in compression. The Raman spectroscopy can also be used for a defect density assessment (mainly point defects) by monitoring the FWHM of the E_2_(high) peak. The values of FWHMs for E_2_(high) were 4.61, 4.83, 3.79, 4.22 and 5.46 cm^−1^ for samples A, B, C, D and E, respectively. In addition, the intensity of E_2_(high) was strong for samples B and C. Therefore, sample C has the best crystal quality, which coincides with the results of the XRD measurements.

**Figure 6:**
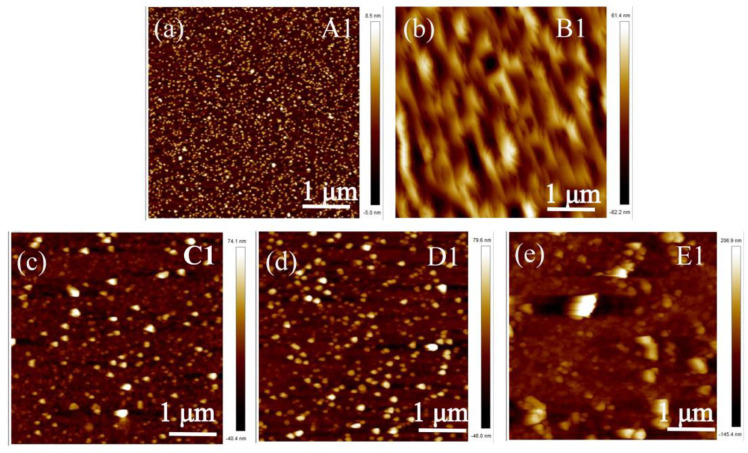
The AFM surface image of samples A1 (**a**), B1 (**b**), C1 (**c**), D1 (**d**) and E1 (**e**).

**Figure 7:**
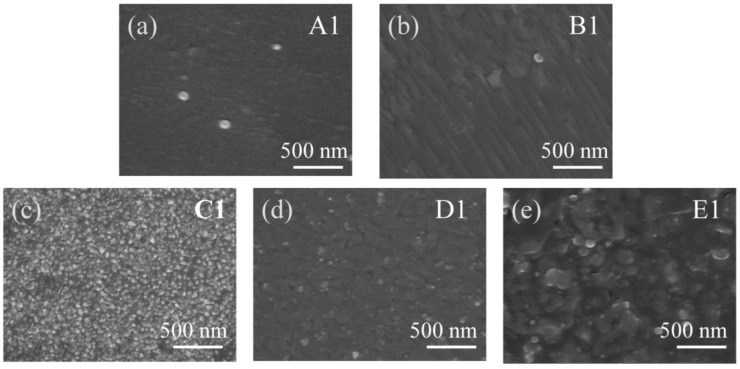
The SEM surface image of samples A1 (**a**), B1 (**b**), C1 (**c**), D1 (**d**) and E1 (**e**).

**Figure 8:**
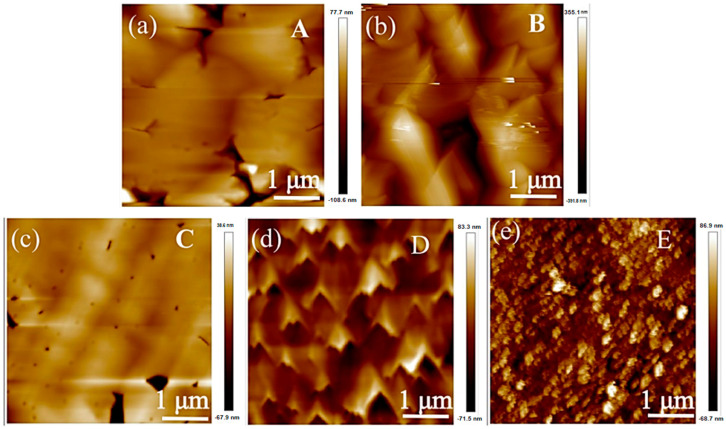
The AFM surface images of samples A (**a**), B (**b**), C (**c**), D (**d**) and E (**e**).

**Figure 9:**
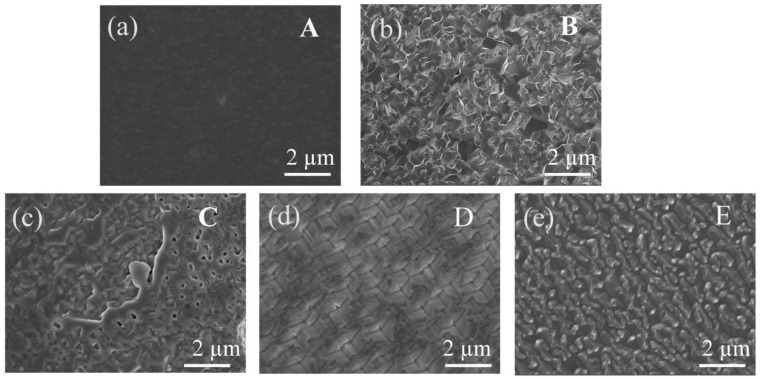
The SEM surface images of samples A (**a**), B (**b**), C (**c**), D (**d**) and E (**e**).

To understand the influence of the annealed MS buffers, AFM and SEM images were performed. Figure 6a shows the surface image of sample A1. The strain at the AlN/sapphire interface prevented the entire columns from twisting, and each column split into a number of small, irregularly shaped domains. It can be seen that the surface of the AlN thin film is composed of small domains, and the surface morphology is relatively smooth, as shown in the SEM micrograph in Figure 7a. Then, in Figure 7b–e, after high-temperature annealing, it was found that the small domains on the surface of the HTA AlN films gradually fused, the grain boundaries reduced, the impurities and defects between the grain boundaries reduced, and the crystalline quality improved, but there was a partially decomposed situation in the E1 samples after annealing at 1600 °C, which led to the deterioration of the quality. Due to the solid phase growth at elevated annealing temperatures, the split small domains coalesced, reducing the number of domains. As the annealing temperature increased, the domain boundaries in the field of view disappeared as a result of gradual coalescence, and high-quality AlN films were obtained. The crystalline angle of the islands showed a broad distribution, as indicated by the FWHM shown in Figure 3b. Figure 6b–e show the images of samples B1-E1, respectively. After annealing at 1400 °C, the small islands coalesced as large islands, as shown in Figure 6b. However, as the annealing temperature was further increased, the MS buffer seemed to decompose, and small islands were formed on the surface. After annealing at 1600 °C, there were large islands with a low density left on the surface. Figure 8a–e show the AFM images of samples A-E, respectively. The surface of samples A and B were composed of large islands that coalesced to form continuous films. Sample E showed an uncoalescing surface with high-density islands, which was due to the low density of nucleation sites resulting from the decomposition of the MS buffer at a high annealing temperature. Although samples C and D formed continuous films, the surface of sample D showed typical triangle characteristics due to the anisotropic growth along different directions, and all triangles exhibited similar geometries with apex angles of about 30°. The stepped edges of the triangles were formed by {20–23} facets that might have been derived from the slip surfaces of stacked layer faults [29]. The surface morphology of sample D in Figure 9d showed the same outcome, where numerous triangles appeared on the surface. From the above test results, the annealing and growth conditions of the E samples could yield high-quality AlN films with smooth surfaces. The SEM surface topography of the HVPE-AlN films in Figure 9a–e were in agreement with the AFM results.

To clarify the influence of the annealing temperature of the MS buffer on the anisotropy of the epilayers, the anisotropy of the XRD linewidth was calculated using the following formula [43]:

$$\rho =\frac{\left|{FWHM}_{11\bar{2}3}-{FWHM}_{1\bar{1}00}\right|}{\left|{FWHM}_{11\bar{2}3}+{FWHM}_{1\bar{1}00}\right|}$$

The anisotropy for both epilayers and MS buffers is shown in Table 3. The anisotropy of the epilayers was less than that of the corresponding MS buffers. It should be noted that as the annealing temperature increased, the anisotropy of the epilayers decreased and reached the minimum at the annealing temperature of 1500 °C. Thereafter, the anisotropy increased again with an increasing annealing temperature, possibly due to the decomposition of MS buffers. M. McLaurin et al. [44] theoretically analyzed that basal plane stacking faults are the main source of anisotropy in the width of the rocking curve, which is a view supported by the report of Zhao et al. [29]. The width of the XRC decreases in all directions after the HTA, indicating a decrease in the defect density and an improvement in the quality of the crystals. These results are in agreement with the AFM measurements shown in Figure 7. Therefore, the reduced anisotropy implies that HTA can effectively reduce the stacking layer error density and improve the quality of (11–22) AlN films, while the individual anisotropy of the re-grown semipolar films is then further reduced via HVPE, and the surface semipolar AlN films are used to efficiently improve the crystalline quality of AlN films with a combination of HTA and HVPE.

## 4. Conclusions

The influence of the annealing process of MS AlN buffers on the crystal quality of AlN epilayers grown via HVPE was investigated. The MS (11–22) AlN buffers on m-plane sapphire were annealed at high temperatures of 1400–1600 °C, and the AlN epilayers were grown at 1450 °C. The crystal quality of the epilayers was significantly improved compared to the MS counterparts. In addition, as the annealing temperature increased, the crystal quality of both MS buffers and AlN epilayers increased first and then decreased due to the decomposition of MS buffers. The increase in the annealing temperature below 1500 °C also resulted in a decrease in the anisotropy of the epilayers. Overall, the HTA and HVPE regrowth processes showed great potential for improving the crystal quality of AlN semipolar layers, providing a promising approach for the realisation of highly efficient optoelectronic devices.

## Figures and Tables

**Table 1:** The study of aluminium nitride films prepared using the MS-HVPE method.

Author References	Substrate	Sputtering Temperature (°C)	Variant	Annealing Temperature (°C)	HVPE Growth Temperature (°C)	(002) FWHM (Arcsec)	(102) FWHM (Arcsec)
Di-Di Li et al. [30]	Sapphire	650	V/Ⅲ	1500	500	64	648
Shiyu Xiao et al. [31]	NPSS	600	Growth temperature	1700	1450–1550	102	219
Jun Huang et al. [32]	Sapphire	650	Annealing temperature and time	1400–1550	1500	28	418
Shiyu Xiao et al. [33]	μPSSs	600	Two-step growth	1700	1500–1600	110	390
Yudai Nakanishi et al. [34]	NPSS		HVPE/ MOVPE	1700	1500	4.7 × 10^8^ cm^−2^	

**Table 2:** The names and characteristics of the samples.

HVPE-AlN Samples	Annealing Temperature (°C)	HTA-AlN Buffer Layer
A	0	A1
B	1400	B1
C	1500	C1
D	1550	D1
E	1600	E1

**Table 3:** The anisotropic values for both epilayers and MS buffers.

	w/o Annealing	1400 °C	1500 °C	1550 °C	1600 °C
HTA MS buffer	0.30	--	0.14	0.10	0.13
HVPE-AlN	0.19	0.08	0.04	0.06	0.31

## Data Availability

The data that support the findings of this study are available from the leading author, X.Y., upon reasonable request.
